# A Genetic Cascade of *let-7-ncl-1-fib-1* Modulates Nucleolar Size and rRNA Pool in *Caenorhabditis elegans*

**DOI:** 10.1371/journal.pgen.1005580

**Published:** 2015-10-22

**Authors:** Yung-Hsiang Yi, Tian-Hsiang Ma, Li-Wei Lee, Pey-Tsyr Chiou, Po-Hsiang Chen, Ching-Ming Lee, Yu-De Chu, Hsiang Yu, Kuei-Ching Hsiung, Yi-Tzang Tsai, Chi-Chang Lee, Yu-Sun Chang, Shih-Peng Chan, Bertrand Chin-Ming Tan, Szecheng J. Lo

**Affiliations:** 1 Molecular Medicine Research Center, Chang Gung University, TaoYuan, Taiwan; 2 Department of Biomedical Sciences, College of Medicine, Chang Gung University, TaoYuan, Taiwan; 3 Graduate Institute of Biomedical Sciences, College of Medicine, Chang Gung University, TaoYuan, Taiwan; 4 Graduate Institute of Microbiology, College of Medicine, National Taiwan University, Taipei, Taiwan; 5 Genome and Systems Biology Degree Program, National Taiwan University and Academia Sinica, Taipei, Taiwan; The University of North Carolina at Chapel Hill, UNITED STATES

## Abstract

Ribosome biogenesis takes place in the nucleolus, the size of which is often coordinated with cell growth and development. However, how metazoans control nucleolar size remains largely unknown. *Caenorhabditis elegans* provides a good model to address this question owing to distinct tissue distribution of nucleolar sizes and a mutant, *ncl-1*, which exhibits larger nucleoli than wild-type worms. Here, through a series of loss-of-function analyses, we report that the nucleolar size is regulated by a circuitry composed of microRNA *let-7*, translation repressor NCL-1, and a major nucleolar pre-rRNA processing protein FIB-1/fibrillarin. In cooperation with RNA binding proteins PUF and NOS, NCL-1 suppressed the translation of FIB-1/fibrillarin, while *let-7* targeted the 3’UTR of *ncl-1* and inhibited its expression. Consequently, the abundance of FIB-1 is tightly controlled and correlated with the nucleolar size. Together, our findings highlight a novel genetic cascade by which post-transcriptional regulators interplay in developmental control of nucleolar size and function.

## Author Summary

Among the RNA/protein bodies within the nucleus, nucleoli are essential factories for ribosome production and assembly. The size and morphology of the nucleolus is thus a cytological manifestation of protein biosynthesis and is closely coordinated with cell biology and even malignancy. However, without membrane delimitation, the principles that define nucleoli size are poorly understood. *Caenorhabditis elegans* represents an ideal model to address this question owing to distinct tissue distribution of nucleolar sizes and a mutant, *ncl-1*, which exhibits larger-than-normal nucleoli. We report here a genetic cascade of microRNA *let-7* and translation repressor NCL-1, which tightly controls abundance of FIB-1/fibrillarin. This network ultimately contributes to developmental control of nucleolar size and function.

## Introduction

Among the RNA/protein bodies within the nucleus, nucleoli bear the essential function of being the factories for ribosome subunit production and assembly, a stress sensor for cell cycle control, as well as a site for hepatitis D virus (HDV) replication and adenovirus-associated virus (AAV) assembly [1–3]. The size and morphology of the nucleolus is a cytological manifestation of ribosome biogenesis and therefore protein biosynthesis and is closely coordinated with cell growth and development [4]. Accordingly, these attributes sometimes are also physiological indicators of cell cycle, cancer growth and malignancy as well as stem cells differentiation and pluripotency [5, 6]. However, without membrane delimitation, the principles that define nucleoli size and shape are poorly understood. Furthermore, spatiotemporal regulation of nucleolar size and output, particularly in coordination with development and in non-dividing cells, are not fully characterized.

*Caenorhabditis elegans* represents an exploitable model for further interrogating nucleolus biology owing to distinct distribution of nucleolar sizes in different cell types. A *C*. *elegans* mutant, *ncl-1*, described as a recessive mutation with enlarged nucleoli in nearly all cells of the worm [7, 8], has this phenotype consistent with its role as a suppressor of rRNA biosynthesis. *C*. *elegans ncl-1* phenotypes can be rescued by its *Drosophila* homolog, *brat* [9, 10]. Mutations in the fly *brat* gene have a similar phenotype to the defect of *ncl-1* mutants in *C*. *elegans*, affecting nucleolar size. In addition, *brat* mutants induce brain tumor formation [11]. These homologous proteins belong to a TRIM/RBCC/NHL (NCL-1, HT2A, and LIN-41] family characterized by the presence of a RING domain, a B-box zinc finger, and a coil-coiled domain [12, 13]. Because its lack of an RNA binding motif, Brat protein was previously shown to associate with the 3’UTR of the *hunchback* transcript in partnership with two RNA-binding proteins Pumillio (PUF) and Nanos (NOS), and suppress expression of Hunchback protein at the translational level [14].

In this study, we dissected the molecular mechanism through which NCL-1 controls nucleolar size and function, and pinpointed fibrillarin—the rRNA 2’-O-methyltransferase and pre-rRNA processing factor [1, 15–17]–as a downstream effector. Further, this regulation is dynamically coordinated with development as part of a functional axis driven by *let-7*, a critical developmental regulator of heterochronic development in worms and flies [18–20] and of cancer formation and stem cell maintenance in the mammals [21].

## Results

### Suppression of nucleolar size and rRNA expression by NCL-1 is associated with nucleolar protein FIB-1

**Fig 1:**
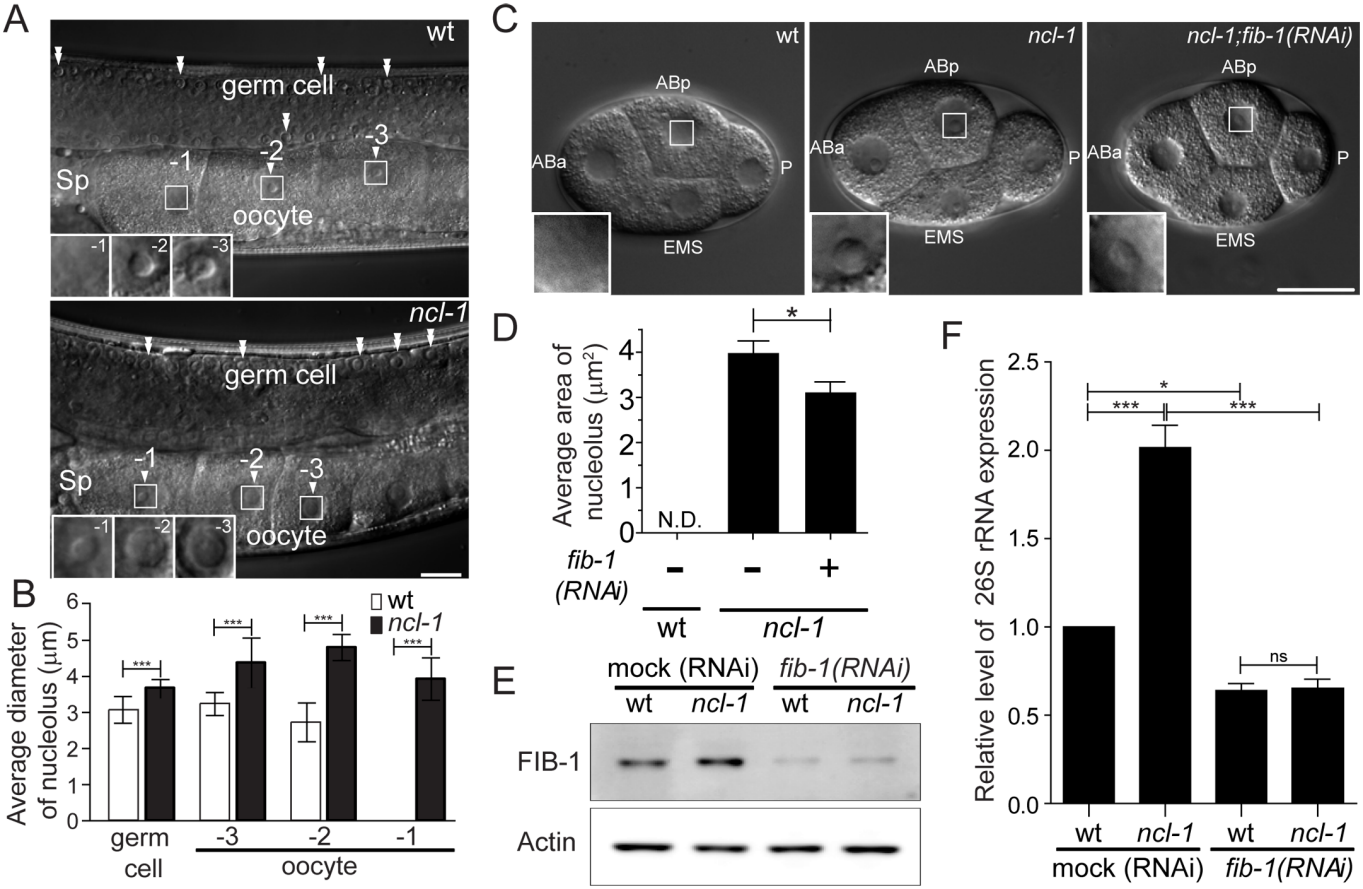
NCL-1 acting as a restriction factor for nucleolar size and rRNA expression is associated with nucleolar protein, FIB-1. (A) Differential contrast interference (DIC) microscopy of the gonads of the wild-type (wt) or *ncl-1(e1942)* worms. Numbers mark the different oocytes and arrowheads indicate the location of nucleoli. Double arrowheads indicate the nucleoli of germ cells. Insets at lower left are enlargement from the square areas of oocytes. Sp, spermatheca. Scale bar, 20 μm. (B) Diameters of the nucleoli in the gonadal cells, as shown in (A), were quantitatively determined. Asterisks signify differences between the two worms: ***P < 0.0001; *n* = 8–14 gonad arms. (C) DIC microscopy of the blastomeres of wild-type (wt) and the *ncl-1* embryos, with or without *fib-1* RNAi. Each blastomere is indicated as ABa, ABp, EMS and P. Insets represent enlarged versions of the boxed regions of ABp cells to highlight the nucleoli. Scale bar, 20 μm. (D) Quantitative representation of the results shown in (C), which illustrates the distribution of nucleolar areas in the four blastomeres. Asterisk signifies difference between the indicated strains: *P < 0.05; *n* ≥ 31 embryos. (E) Knockdown of *fib-1* was done in the indicated worms. The expression of Actin (lower panel) and the endogenous FIB-1 (upper panel) were examined by Western blot analysis. (F) RT-qPCR analysis of 26S rRNA expression in the indicated strains of worms as shown in (E) *P < 0.05; ***P < 0.001; ns, no significant; n = 3.

Although abundantly expressed in the gonads of *C*. *elegans* [9], the effect of *ncl-1* on the nucleoli of germ cells was not characterized. In intact gonads of wild-type (N2) young adult worms, nucleolar structure is nearly absent in the -1 oocyte, which is immediately adjacent to the spermatheca (Fig 1A, upper panel). In contrast, the nucleolus was readily detectable in the -1 oocyte of *ncl-1*(*e1942*) mutant (Fig 1A, lower panel). While nucleoli were evident in the germ cells and the -3 and -2 oocytes of both worms, *ncl-1* worms exhibited considerably larger average nucleoli size ranging from 119% to 176% of wild-type diameter (Fig 1A and 1B). Profiling of the *ncl-1* mRNA expression by RT-qPCR revealed a progressive decline in mRNA abundance from the embryo to and throughout the four larva stages, followed by subsequent up-regulation in the adult (S1 Fig). This developmental stage-specific expression is consistent with previous *in situ* immuno-staining of NCL-1 that demonstrated its expression in the proximal gonad and early embryos and the subsequent gradual disappearance in the late stages of embryos [9]. Further, this expression is in line with the non-detectable to small sizes of nucleoli in the -1 oocyte and early embryos (Fig 1C, left panel), supporting the notion that NCL-1 is a negative regulator of nucleolar size.

We also examined nucleolar morphology in worms devoid of functional *fib-1*. Consistent with its significance, *fib-1* mutation led to lethality [22]; we thus characterized *fib-1* mutant larvae (L1 stage) and found that nucleoli therein displayed size reduction (S2 Fig). To next determine if FIB-1/fibrillarin is involved in the nucleolar appearance and size, we depleted *fib-1* in *ncl-1(e1942)* worms by RNAi feeding and measured the nucleolar size. The increase in nucleolar size in the blastomeres of *ncl-1(e1942)* worms (Fig 1C, middle panel) was significantly reversed by *fib-1* abrogation as shown by image analysis (Fig 1C, right panel, and 1D). This observation supports a notion that the amount of FIB-1 expression is directly associated with the control of nucleolar size by NCL-1. Moreover, Western blot and RT-qPCR analyses showed that worms expressing a greater amount of FIB-1 generally had a higher level of rRNA abundance (Fig 1D and 1F). Conversely, knockdown of FIB-1 led to an overall reduction in the rRNA levels, further indicating a positive role of FIB-1 in this functional regard.

### NCL-1 is a suppressor of FIB-1 expression

**Fig 2:**
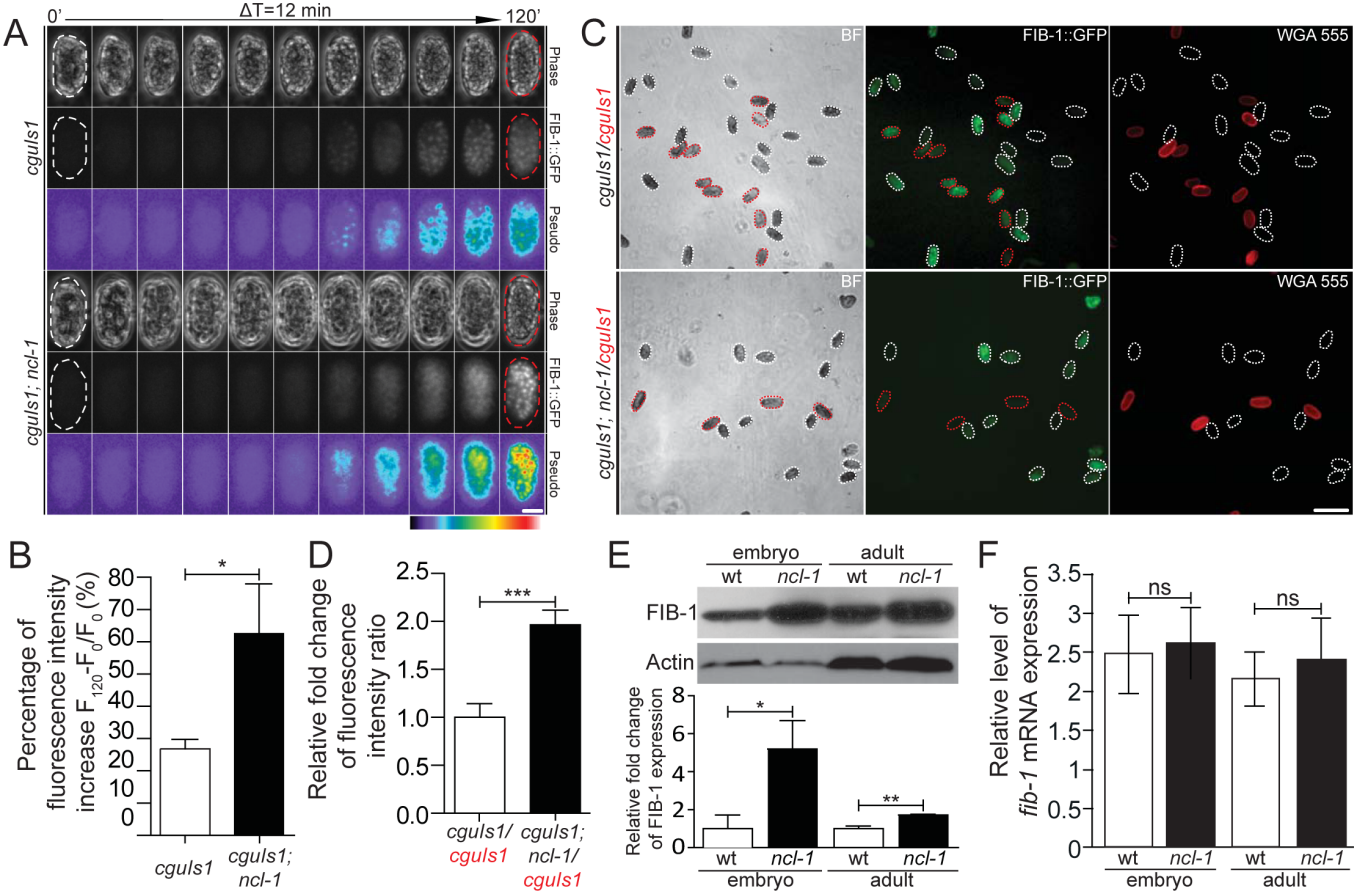
NCL-1 is a negative regulator of FIB-1 expression. (A) Time-lapse imaging of embryos from the two indicated transgenic worms during early development, taken every 12 minutes. Both phase-contrast and green fluorescence (FIB::GFP) images are shown, with pseudo-colors depicting the degree of fluorescence intensity as indicated by a color bar. Scale bar, 20 μm. (B) Average fluorescence levels were quantitatively determined from the images in (A). The bar graph shows the percentage of fluorescence intensity increase between the initial and end-point time-lapse images for the indicated strains. *P < 0.05; *n* = 20–23 embryos. (C) Fluorescence images shown the FIB-1::GFP expression in different transgenic embryo pairs. Embryos of the indicated strains were labeled either with or without WGA 555 prior to mixture at an equal ratio (labeled strains are marked in red). Representative images are shown, with white and red dotted contours respectively marking WGA-negative and WGA-positive embryos (“BF”, bright field). Scale bar, 100 μm. (D) Quantitative image analysis for FIB-1::GFP expression in embryos shown in (C), illustrating the ratios of average fluorescence intensity between the indicated strains. Asterisk signifies the difference: ***P < 0.0001; *n* = 129–222 embryos. (E) and (F) Western blot and RT-qPCR analyses of FIB-1/*fib-1* mRNA and Actin/*actin* mRNA (as control) in the wild-type (wt) or *ncl-1* animals, at the embryo or adult stage. Relative levels of normalized FIB-1/*fib-1* mRNA expression are quantified and presented below. The bar graph depicts means ± S.E.M.; *P < 0.05; **P < 0.005; ns, no significance; *n* = 3–5.

To examine whether NCL-1-mediated nucleolar size alternations is through the regulation of FIB-1 expression, we generated a pair of transgenic worms that express FIB-1::GFP chimeric protein in both the N2 and *ncl-1* backgrounds [respectively designated as *cguIs1* (strain SJL1) and *ncl-1(e1942); cguIs1* (strain SJL14), see S1 Table]. Time-lapse fluorescence microscopy of embryos was performed to trace the level of GFP expression during early stages, and showed progressively higher GFP signals (Fig 2A and S1–S3 Movies). Dynamic up-regulation of GFP levels was more prominent in the *ncl-1(e1942); cguIs1* embryos (62.8%) than in *cguIs1* (26.9%) (Fig 2B). Random collections of embryos from both transgenic worms were further examined to quantify the GFP intensity of each embryo in the same field (Fig 2C) and subsequently revealed that the embryos in the absence of NCL-1 exhibited higher levels of FIB-1::GFP (about 2 fold) (Fig 2D). Further expression analyses consistently showed elevated levels of FIB-1 in *ncl-1(e1942)* embryos (5.2 fold) and adult worms (1.7 fold) (Fig 2E). Unexpectedly, RT-qPCR analysis revealed comparable levels of *fib-1* mRNA in wild type and *ncl-1(e1942)* in embryo and adult stages (Fig 2F). Taken together, these findings indicate that *ncl-1* is an upstream negative regulator of *fib-1* expression at the post-transcriptional/translational stage.

### NCL-1 cooperates with PUF and NANOS to modulate fib-1 mRNA translation

**Fig 3:**
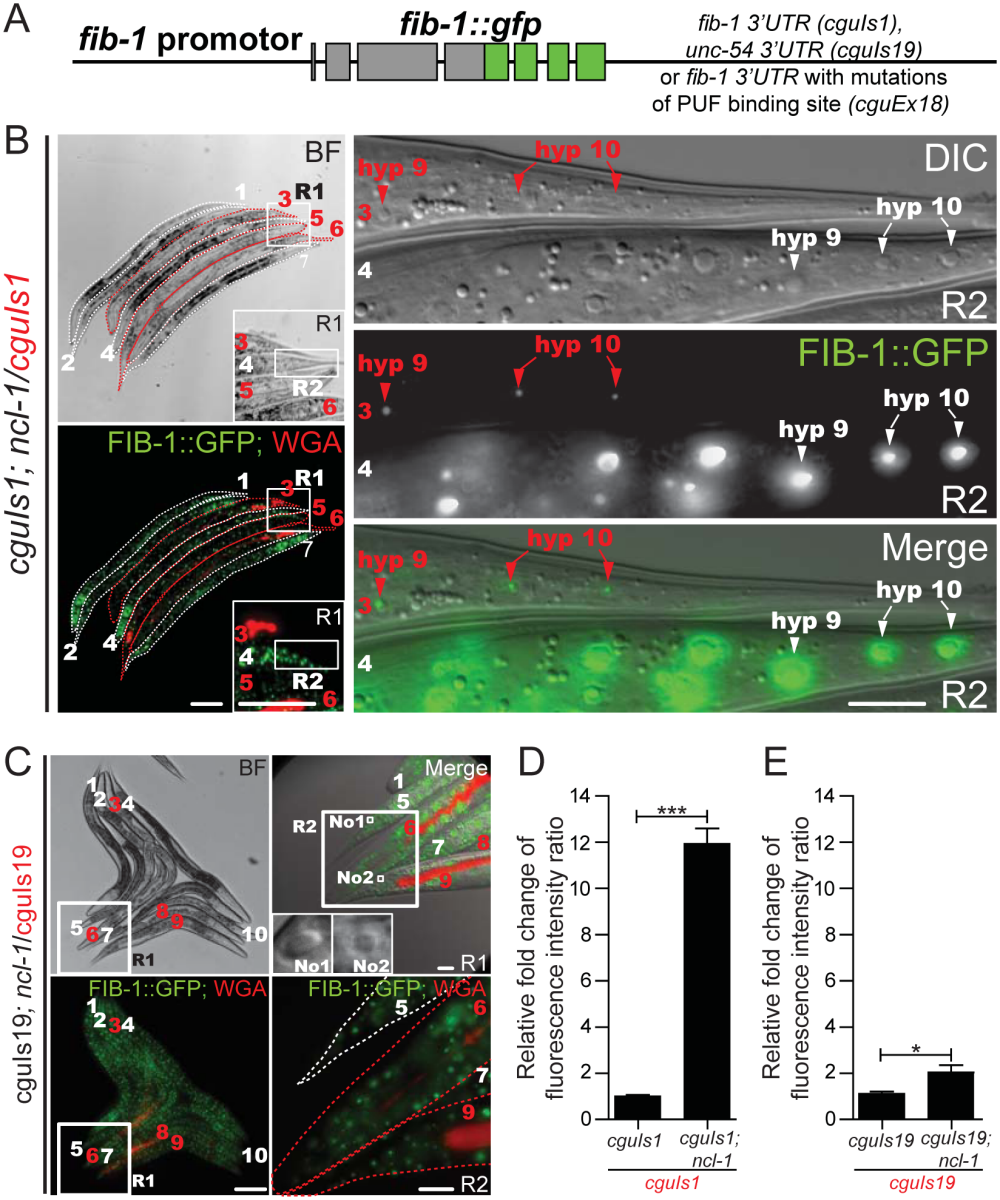
The 3’ UTR of *fib-1* is under the control of NCL-1. (A) Schematic for a FIB-1::GFP transgene construct that harbors 3' UTR sequence from either the *fib-1* or *unc-54* gene, or with mutated PUF binding site. (B) Before image acquisition, worms of different strains were first labeled with or without WGA 555 prior to mixture at an equal ratio. Expression of the *fib-1* 3' UTR reporter (FIB-1::GFP) was examined in the N2 (*cguIs1*; WGA-positive and indicated by numbers and contours in red) and *ncl-1* mutant (*ncl-1(e1942); cguIs1*; WGA-negative and indicated by numbers and contours in white) backgrounds. Insets in the left panels represent enlarged versions of the boxed regions (R1); images on the right denote magnified versions of the boxed regions in the corresponding images on the left (R2), and represent the tail hypodermis. Red and white arrowheads pinpoint the hyp9 and hyp10 cells of respectively the *cguIs1* and *ncl-1(e1942)*; *cguIs1* worms. Scale bar, 100 μm in images on the left and 10 μm in images on the right. (C) Comparison of FIB-1::GFP expression of a pair of worms carrying a reporter with *unc-54* 3' UTR (*cguIs19* and *ncl-1(e1942); cguIs19*, respectively WGA-positive and WGA-negative, and indicated by numbers in red and in white). Image on the upper right denotes a magnified version of the boxed region in the corresponding image on the left (R1) and represents the tail region. Insets of upper right panel represent the nucleolus (labeled with No1 and No2) of tail hypodermis from worm 5 and worm 9, respectively. Note the enlargement of nucleolus in *ncl-1(e1942); cguIs19* but similar fluorescence intensity as *cguIs19* (lower right image, which is an enlarged version of the R2 box region from upper right image). Scale bar: 100 μm (left panels) and 10 μm (right panels). (D)-(E) Quantitative image analysis for FIB-1::GFP reporter expression in the whole worms. Fluorescence in images shown in Fig 3B (the *cguIs1* and *ncl-1(e1942); cguIs1* worm pair containing a full-length of *fib-1* 3' UTR) (D), in Fig 3C (the *cguIs19* and *ncl-1(e1942); cguIs19* worm pair containing the *unc-54* 3' UTR) (E), were quantitatively determined, with ratios between the indicated strains being shown in the bar graph. Asterisk signifies the difference: *P < 0.05; ***P < 0.001; *n* = 148–215 animals.

We next aimed to test whether NCL-1 acts as its fly homologue Brat, which suppresses its target gene at the translational level by binding to the 3' UTR of transcripts [14]. Towards this end, we created two more pairs of transgenic worms [*cguIs2* and *ncl-1(e1942); cguIs2* (strain SJL2/strain SJL15), and *cguIs19* and *ncl-1(e1942); cguIs19* (strain SJL34/strain SJL38), see S1 Table]; SJL2 and SJL15 harbored a plasmid similar to *cguIs1* worms that contains the full-length *fib-1* 3' UTR, while in SJL34 and SJL38 the *fib-1* 3' UTR was replaced with *unc-54* 3' UTR sequence (Fig 3A). In agreement with the above observations, enlarged nucleoli and a significantly increased levels of FIB-1::GFP expression were both evident in the tail hypodermis of *ncl-1(e1942); cguIs1* and *ncl-1(e1942); cguIs2* worms (Fig 3B, top two panels at right, and S3 Fig). In contrast, for the transgene harboring the *unc-54* 3' UTR, *ncl-1* inactivation did not lead to discernable difference in GFP intensity, despite the occurrence of enlarged nucleoli of cells in *cguIs19; ncl-1* transgenic worms (Fig 3C). These observations and the quantitative data for the whole worms (Fig 3D and 3E) strongly support the notion that, rather than being the consequence of altered nucleolus, the suppression of FIB-1 may arise from direct targeting of its 3' UTR by NCL-1.

**Fig 4:**
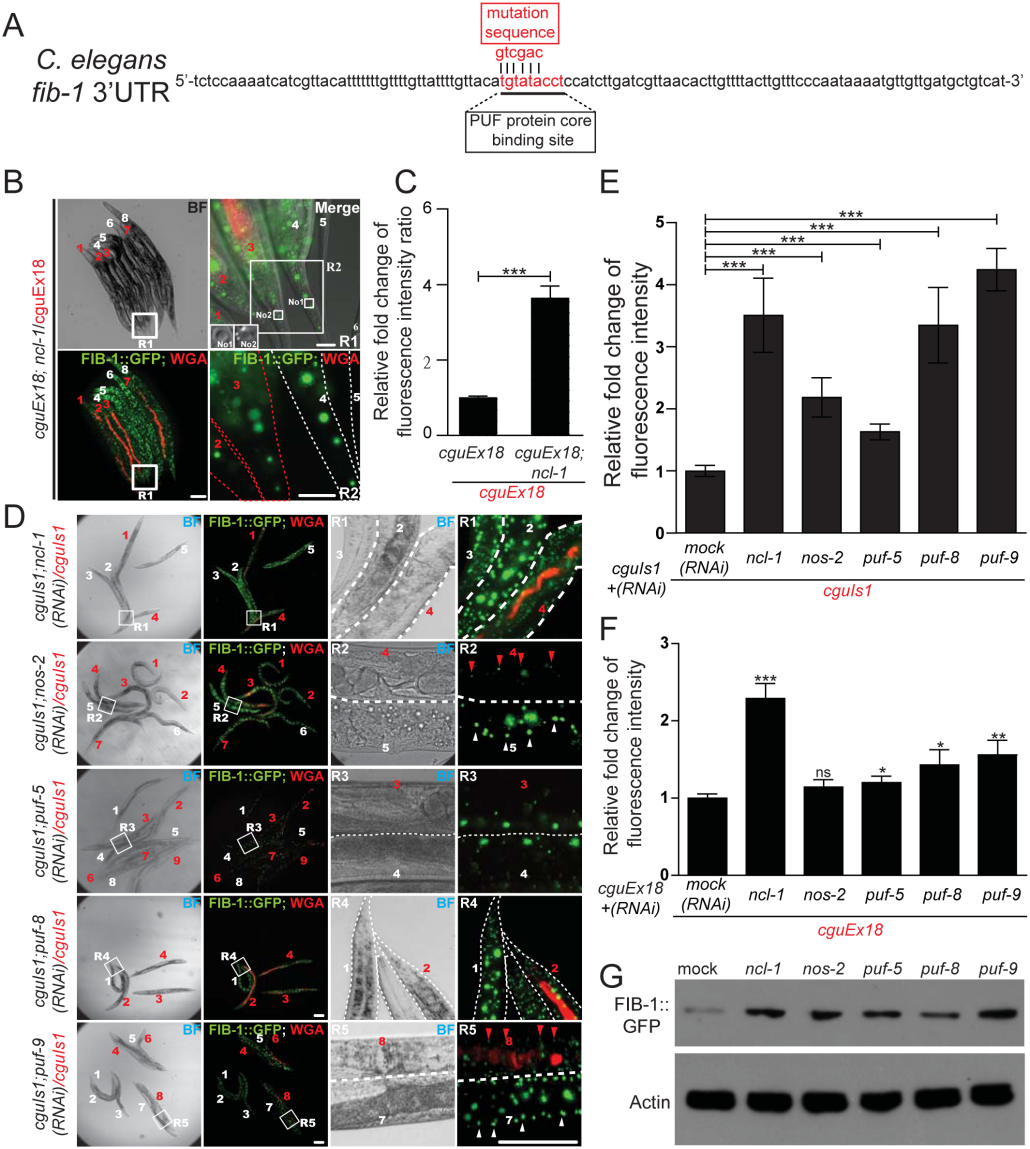
NCL-1 cooperates with PUF and NANOS to modulate FIB-1 expression. (A) Putative PUF target sites within the *fib-1* 3' UTR (underlined). Mutant nucleotide sequence used to generate the altered reporter in *cguEx18* worms is shown above the sequence (mutation sequence). (B) Comparison of FIB-1::GFP expression of a pair of worms carrying a reporter with mutated *fib-1* 3' UTR (*cguEx18* and *ncl-1(e1942); cguEx18)*, respectively WGA-positive and WGA-negative, and indicated by numbers in red and in white). Image on the upper right denotes a magnified version of the boxed region in the corresponding image on the left (R1) and represents the tail region. Insets of upper right panel represent the nucleolus (labeled with No1 and No2) of tail hypodermis from worm 4 and worm 3, respectively. Lower right image is an enlarged version of the R2 box region from upper right image. Scale bar: 100 μm (left panels) and 10 μm (right panels). (C) In Fig 4C (the *cguEx18* and *ncl-1(e1942); cguEx18* worm pair containing the mutated *fib-1* 3' UTR) were quantitatively determined, with ratios between the indicated strains being shown in the bar graph. Asterisk signifies the difference: ***P < 0.001; *n* = 136–156 animals. (D) *cguIs1* worms with RNAi targeting *ncl-1*, *nos-2*, *puf-5*, *puf-8*, or *puf-9* were assessed for FIB-1::GFP expression as in (B). Scale bar: 100 μm (left panels) and 10 μm (right panels). (E) Quantitative image analysis for the results shown in (D), showing the relative ratios of average FIB-1::GFP signals between the indicated worm pairs. The bar graph depicts means ± S.E.M.; ***P < 0.001; *n* = 30–198 animals. (F) Quantitative image analysis for the FIB-1::GFP reporter expression in worm strains derived from *cguEx18*, showing the relative ratios of average FIB-1::GFP signals between the indicated worm pairs. The bar graph depicts means ± S.E.M.; *P < 0.05; **P < 0.01;***P < 0.001; ns, no significance; *n* = 30–198 animals. (G) Expression of the FIB-1::GFP reporter in worms with RNAi targeting the indicated genes was monitored by anti-GFP immunoblotting.

Since Brat mediates its repressive role through other RNA-binding factors, we further tested the roles of *C*. *elegans pumillio* and *nanos* in the translational suppression of *fib-1*. A potentially direct involvement of these RNA-binding proteins was first supported by the sequence analysis of the *fib-1* 3’UTR, which revealed a consensus PUF binding motif (Fig 4A). To demonstrate the link between this 3’UTR element and NCL-1-dependent control, we then generated worms with 3’UTR reporter carrying mutations in the PUF binding sequence (*cguEx18*; Figs 3A and 4A) [23, 24]. Fluorescence microscopy showed that, in comparison to the wild-type reporter (Fig 3B and 3D), this particular transgene exhibited considerably diminished responsiveness to the loss of *ncl-1* (Fig 4B and 4C), giving rise to a lower level of fluorescence intensity. In further support to the roles of the PUF proteins, RNAi knockdown *puf-5*, *puf-8* and *puf-9* and *nos-2* in *cguIs1* worms resulted in the appearance of brighter GFP signals (Fig 4D and 4E). However, such effect of *nos*/*puf* knockdown (*puf-8* and *puf-9* in particular) on the GFP reporter was reduced in the *cguEx18* worms, in which the PUF binding sequence was altered (Fig 4F). Consistently with the *ncl-1* knockdown and mutant worms, immunoblotting showed a rise in FIB-1::GFP abundance in these knockdown worms (Fig 4G). Collectively, these data imply that *ncl-1* may coordinate with *puf-5*, *-8*, *-9* and *nos-2* to act directly on the 3' UTR element of *fib-1*, likely through a similar regulatory mechanism exhibited by *brat*, *pumillio* and *nanos* in the fly [14]. This demonstration of a response element in the *fib-1* 3’UTR and its regulatory relevance would certainly strengthen a specific and direct control mechanism.

### The *let-7-ncl-1-fib-1* pathway controls the nucleolus size and rRNA pool

**Fig 5:**
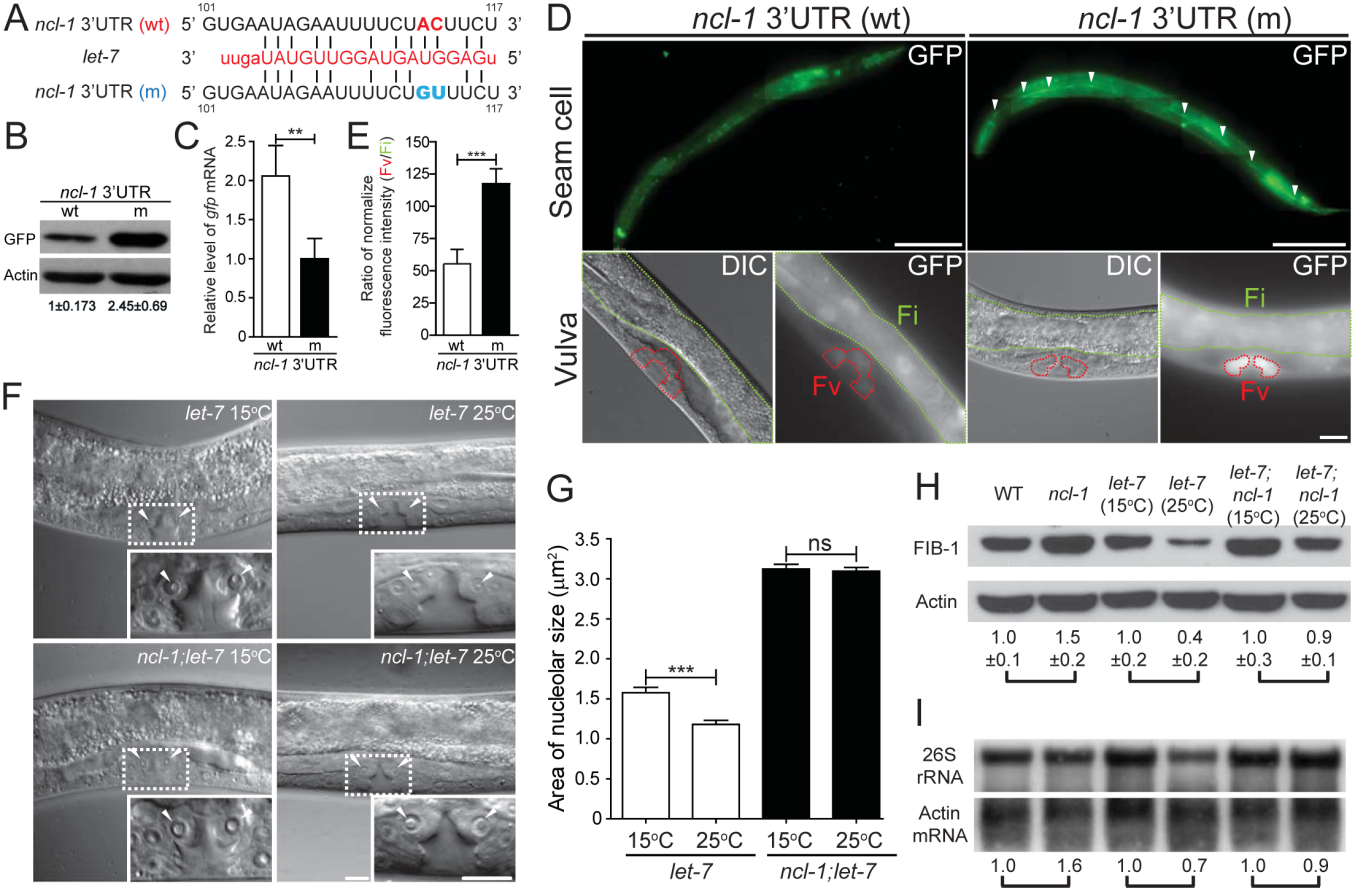
The *let-7-ncl-1-fib-1* pathway controls the nucleolus size and function. (A) Putative miRNA target sites within the *ncl-1* 3' UTR. The alignment on the right indicates the *ncl-1* 3' UTR/*let-7* complement, with numbers denoting sequences relative to the stop codon. GFP reporter construct (driven by *ncl-1* promoter) that contains wild-type 3' UTR sequence (wt) or mutated *let-7* target sites (m) was used to generate transgenic worms. (B) Expression of the GFP protein and mRNA expression in worms carrying the reporter gene with wild-type (wt) vs. mutant (m) *ncl-1* 3' UTR. (A) Immunoblots of GFP reporter and the control Actin in the indicated worms. Numbers below represent relative levels of normalized GFP expression, based on quantified intensity of immunoblotting signals from three independent experiments. (C) Quantitative RT-qPCR analysis of *gfp* mRNA expression in the indicated transgenic worms. **P < 0.01; *n* = 4. (D) Characterization of GFP reporter expression in the seam (top; scale bar of 100 μm) and vulva (bottom; scale bar of 10 μm) cells. Arrowheads point to the seam cells, while red and green contours respectively denote the vulva (Fv) and intestinal (Fi) areas in the bottom images. (E) Quantitative depiction of the ratios of average fluorescence intensity in the indicated strains. Ratios were derived from the vulva (Fv) vs. intestinal (Fi) comparison, as shown in (D). ***P < 0.001; *n* = 46 animals. (F) DIC microscopy of the vulva cells of the *let-7(n2853)* and *let-7(n2853); ncl-1(e1942)* worms, at the permissive (15°C) or non-permissive (25°C) temperature. Insets represent enlarged images of the boxed regions in the corresponding figures. Arrowheads point to the nucleoli of the vulva cells. Scale bar, 10 μm. (G) Quantitative representation of the results shown in (F), illustrating the distribution of nucleolar areas in the vulva. Asterisk signifies difference between the indicated strains: ***P < 0.001; ns, no significance; *n* = 22–36 [for *let-7(n2853)* worms] or 100–110 [for *ncl-1(e1942); let-7(n2853)* worms]. (H) Western blot analysis of FIB-1 and Actin (as control) in the indicated strains. Numbers below represent the relative levels of FIB-1 protein expression (normalized to the control sample of each pair-wise comparison), calculated from five independent experiments. (I) Northern blot analysis of 26S rRNA and *Actin* mRNA expression in the indicated strains of worms as shown in (H). Numbers below represent the relative levels of 26S rRNA, with control sample of each pair-wise comparison being represented as 1.

Since another TRIM/RBCC/NHL family protein, LIN-41, is regulated by *let-7* [13, 25], we tested for the potential involvement of microRNAs in the regulation of NCL-1. Indeed, potential *let-7* and *mir-49* target sequences were identified in the 3' UTR of *ncl-1* (S4 Fig). As one of the best-known and evolutionarily conserved microRNAs [18, 26, 27], *let-7* was selected for further investigation of possible role in *ncl-1* expression. Two transcriptional reporters–*P*_*ncl-1*_::*gfp*::*3' UTR*_*ncl-1*_ and *P*_*ncl-1*_::*gfp*::*3' UTR*_ncl-1(m)_, respectively harboring the wild type and mutated *let-7* presumptive sites (Fig 5A), were constructed to each generate multiple independent integration lines of transgenic worms (S1 Table). Immunoblotting results revealed that transgenic worms bearing *P*_*ncl-1*_::*gfp*::*3' UTR*_*ncl-1*_ (strain SJL8) expressed less GFP than those of *P*_*ncl-1*_::*gfp*::*3' UTR*_ncl-1(m)_ (strain SJL12) (Fig 5B), despite comparable copy numbers and mRNA levels of the transgene between the two strains (Fig 5C). These results thus indicated a loss of responsiveness to *let-7* suppression. In further support to the *let-7*-*ncl-1* link, fluorescence microscopic analysis of the seam cells and vulva, which are known to express *let-7* [28, 29], indeed showed pronounced GFP reporter expression in the context of defective *let-7* targeting (Fig 5D and 5E; S4 and S5 Movies).

**Fig 6:**
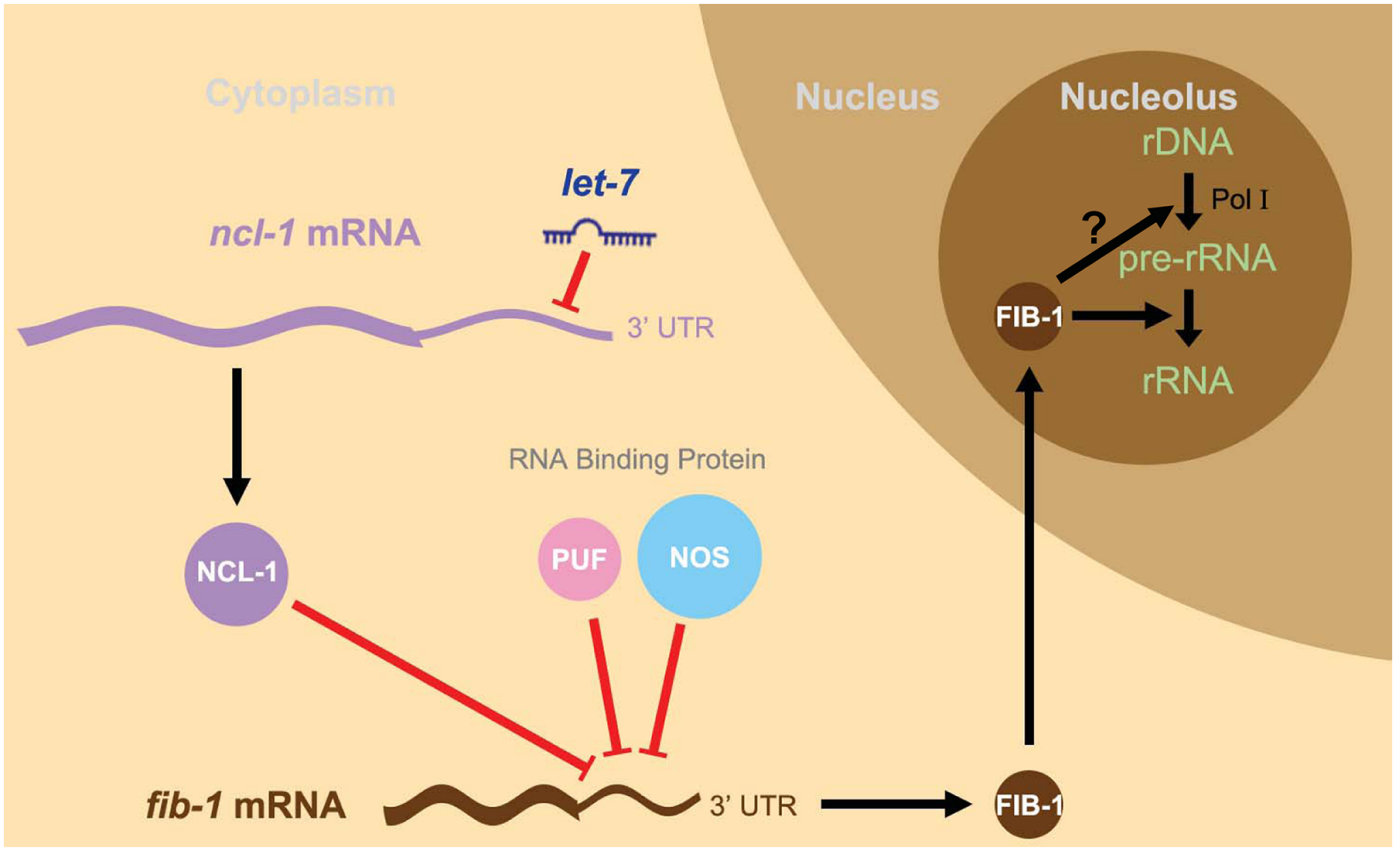
A schematic model of the *let-7-ncl-1-fib-1* circuit and its regulation of nucleolus size and function. Since *let-7* is a heterochronic gene linked to the control of vulva formation in the L4 larva stage, this model depicts a novel *let-7*-driven regulatory cascade—the l*et-7-ncl-1-fib-1* pathway—that regulates the nucleolus size and rRNA expression in the vulva cells. In this context, *let-7* increases in the L4 larva and targets the 3’ UTR of *ncl-1* transcript to suppress NCL-1 translation. In other types of cells with low levels of *let-7*, such as hypodermis for example, NCL-1 may be accumulated and cooperates with two other RNA binding proteins, PUF and NOS, to suppress translation of a nucleolar protein FIB-1 and consequently the size of the nucleolus (see Fig 4B and 4C). However, in the vulva cells in which NCL-1 is down-regulated, a higher abundance of FIB-1 enters the nucleolus to facilitate rRNA processing and likely contributes to enlarged nucleolus exhibited by this particular cell type (see Fig 5F and 5G). Possible FIB-1 action on Pol I activity is not resolved in this study (the question mark in the scheme), although one recent study (Tessarz et al., Nature 505, 564–568, 2014) [47] has shown that FIB-1 impacts Pol I transcription through an epigenetic control.

We next interrogated the significance of *let-7* in the regulation of nucleolar size by assessing vulva cells in the temperature-sensitive, loss-of-function *let-7(n2853)* mutants. Mutants grown at non-permissive temperature (25°C) displayed a significant reduction in nucleolar size in these cells, by 25% as compared to those at permissive condition (15°C) (Fig 5F and 5G). However, such temperature-sensitive nucleolar size alteration was not observed in a double mutant *let-7; ncl-1* (strain SJL39) (Fig 5F and 5G), implying that *let-7* acts upstream of *ncl-1* transcript to directly suppress NCL-1 translation and regulate nucleolar sizes of the vulva cells. We further verified the link of *let-7* to NCL-1-mediated regulation by assessing downstream FIB-1 expression and rRNA abundance in *let-7(n2853)* and *let-7(n2853); ncl-1(e1942)* worms. To this end, expression profiling revealed higher amounts of both FIB-1 (Fig 5H) and ribosomal RNA species (Fig 5H and 5I and S5 Fig) in *let-7(n2853)* worms grown at 15°C vs. 25°C, in contrast to a lack of discernable differences in the *let-7(n2853); ncl-1(e1942)* worms between these rearing temperatures (Fig 5H and 5I, and S5 Fig). Such loss of phenotypes in the *ncl-1(e1942)* background is in agreement with *let-7*-*ncl-1* interaction and functional antagonism. Based on these findings, we hypothesize that the genetic circuit of *let-7-ncl-1-fib-1* constitutes a critical determinant in the regulation of nucleolar size and rRNA pool (Fig 6).

## Discussion

*let-7* is known as a critical regulator of heterochronic development in worms and flies [18, 29]. Our studies outlined for the first time a genetic cascade through which the coordinated actions of *let-7* and NCL-1 modulate the expression of a major nucleolar protein FIB-1, thereby fine-tuning the size and function of the nucleolus (Fig 6). This circuit of *let-7*-*ncl-1*-*fib-1* and nucleolus size may represent an adaptive mechanism that couple cellular protein production capacity to the metabolic state of individual cell types. Interestingly, in a recent genome-wide RNAi-based screening for molecular networks underlying nucleolus size regulation in *Drosophila*, both *brat* and *fib* were identified [4], substantiating the possibility that these factors constitute a conserved core of regulatory network. Moreover, Vogt *et al*. has demonstrated that nucleolus maturation during early embryonic development in mice is dependent on the pluripotency factor LIN28 [30], which is known as an essential regulator of *let-7* biogenesis [19, 20, 31]. Intriguingly, Chan and Slack have also shown that ribosomal protein RPS-14 is able to modulate *let-7* function [32], which hints at the possibility for a feedback regulation between *let-7* and nucleolar dynamics. Our work thus contributes to these findings by reinforcing the relevance of hierarchical organization of post-transcriptional regulators in the fundamental process of nucleogenesis. As FIB-1 expression in *C*. *elegans* is also regulated by the *die-1* and *let363/TOR* pathways [33, 34], our findings further support the notion that intricate integration of multiple mechanisms underpins nucleolus integrity.

NCL-1 is a member of TRIM/RBCC-NHL protein family, which has been implicated in the regulation of tumor suppression, cell growth, and cell differentiation. In *Drosophila* larval neuroblasts (stem cell-like precursors), the *Brat* homologue is distributed to only one daughter cell through asymmetric cell division and acts as an inhibitor of its self-renewal through post-transcriptional suppression of Myc expression. In *Brat* mutant, both daughter cells grow and lead to the formation of larval brain tumor [35]. Similarly, the mammalian homologue TRIM3 has been reported as a tumor suppressor in human glioblastoma (GBM), a highly malignant human brain tumor, through its suppression on Myc [36]. Our study complements these findings on the NCL-1 homologues and further provides significant insight into understanding how microRNA cooperates with TRIM/RBCC-NHL proteins to suppress tumor formation.

Despite the prevalent requirement for proper maintenance of nucleolus size, our data did not exclude the possibility that the NCL-1-dependent control mechanism may have tissue- and developmental stage-specific relevance. First, while elevated FIB-1::GFP expression was robustly observed in the *ncl-1* mutant, the extent to which it was up-regulated was varied between cells/tissues. A strong evidence for this phenotype is shown in Fig 3B, in which we observed variation in nucleolar size changes between hyp 9 and hyp 10 cells. Second, and perhaps more intriguingly, even in the absence of putative PUF binding site, loss of *ncl-1* led to a prominently up-regulated GFP reporter expression in the head region of the *cguEx18* worms (Fig 4B). This observation of differential regulation thus implies that 1) there is additional *cis*-acting element(s) in the *fib-1* 3' UTR, through which a yet unknown protein mediates brain-specific expression suppression, and/or 2) NCL-1 may functionally cooperates with other neuronal RNA-binding protein(s) to exert a context-dependent regulation of *fib-1*. This possibility of a modular organization of NCL-1-based regulatory network, as well as its developmental implications, may be further resolved by genetic screens and/or biochemical characterization of NCL-1-interacting factors.

## Materials and Methods

### Strains and mutant alleles of *C*. *elegans*

N2 Bristol *C*. *elegans* (used as a wild-type animal control) and mutant strains were obtained from Caenorhabditis Genetic Center (CGC, Minnesota). Alleles of the mutants are as follows: *ncl-1*(*e1942*) *III*, *fib-1*(*ok2527*) *V*, *unc-119*(*ed3*) *III* and *let-7*(*n2853*) *X*. Transgenic worms generated in this study are listed in S1 Table. Five additional strains for this study were generated by the following crosses: SJL14 *ncl-1(e1942); cguIs1*, SJL15 *ncl-1(e1942); cguIs2*, SJL38 *ncl-1(e1942); cguIs19*, SJL39 *let-7(n2853); ncl-1(e1942)* and SJL118 *ncl-1(e1942); cguEx18*. Worms were cultured at 15°C or 20°C on NGM plate (1.7% agar, 2.5 mg/mL peptone, 50 mM KH2PO4 pH 6.0, 25 mM NaCl, 5 mg/mL cholesterol, 1 mM MgSO4, 1 mM CaCl2) with fresh *Escherichia coli* OP50 as food source [37], and synchronized by the protocol with alkaline hypochlorite treatment and the resulting eggs were seeded onto NGM agar plates [38]. Temperature-sensitive mutants (*let-7*(*n2853*) *X* and SJL39 *let-7(n2853); ncl-1(e1942)*) were maintained at 15°C and shifted to 25°C at the larval 1 (L1) stage and harvested in L4 stage.

### Plasmid constructions

Plasmids containing the genomic region of *fib-1*, including the promoter and 3' UTR of *fib-1*, were constructed from the PCR amplified DNA fragment from the *C*. *elegans* operon CEOP5428 [39]. The *gfp* gene was inserted into the last codon of *fib-1* open reading frame to obtain a translational reporter, *P*_*fib-1*_::*fib-1*::*gfp*::*3' UTR*_*fib-1*_, which encodes a fusion protein of FIB-1::GFP. Another derivative *P*_*fib-1*_::*fib-1*::*gfp*::*3' UTR*_*unc-54*_ was generated by replacing the *fib-1* 3' UTR with *unc-54* 3' UTR sequence, and the construct of P_*fib-1*_::*fib-1*::*gfp*::3' UTR_fib-1(m)_ was done by site-direct mutagenesis of PUF binding site. To construct two transcriptional reporters, the 1.0-kb promoter and 3' UTR of *ncl-1* were cloned into a vector to then generate *P*_*ncl-1*_::*gfp*::*3' UTR*_*ncl-1*_ and *P*_*ncl-1*_::*gfp*::*3' UTR*_ncl-1(m)_, which differ in the *let-7* presumptive targeting sites.

### Worm transformation (microinjection and bombardment)

Germ line transformation by microinjection was performed as described by Mello and Fire [40]. Plasmids at the concentration of 100 ng/μl were injected into young adult N2 worms. An integrated line containing the plasmid of *P*_*fib-1*_::*fib-1*::*gfp*::*3' UTR*_*fib-1*_ in about a hundred copies (determined by RT-qPCR) was first obtained in the wild-type background (designated as SJL1 *cguIs1*). A male of *cguIs1* was then crossed with *ncl-1(e1942)* hermaphrodites, and GFP positive worms were selected. This was followed by hermaphrodite selfing to generate a homozygote worms [SJL14 *ncl-1*(*e1942*); *cguIs1*]. The same method was used to generate the other integration lines (see S1 Table), whereas strains of SJL6 to SJL12 (S1 Table) were obtained by the bombardment method [41].

### RNAi treatment

The RNAi library was obtained from Julie Ahringer's group [42–44]. Bacteria clones producing double-stranded RNA to each target gene were grown in LB broth containing ampicillin and tetracycline for 7 to 8 hrs, and subsequently induced to produce double-stranded RNA by 1 mM isopropyl β-D-1-thiogalactopyranoside (IPTG) for 2 hrs. Concentrated bacteria were then seeded on RNAi plates (NGM agar, 1 mM IPTG, 100 mg/ml ampicillin, and 5 mg/ml tetracycline), onto which synchronized L1- L2 stage worms were placed and cultured for 36 hrs at 25°C. Young adult worms were collected for microscopy, RT-qPCR, and/or Western blot analyses.

### Western blot

Protein extracts from embryos or worms at L4 or young adult stage were prepared by sonication and separated on 10% or 15% SDS-PAGE and transferred onto polyvinylidene fluoride (PVDF) membranes. Blocked membranes were then incubated with anti-FIB-1 (1:2,000 dilution, Santa Cruz) or anti-Actin (1:200,000 dilution, Millipore) antibody overnight at 4°C, and subsequently probed with secondary antibody-horseradish peroxidase conjugate (1:5,000 dilution, Sigma). Signals were detected with the ECL Western blot detection system (Thermo Scientific Inc., Waltham, MA).

### RT-qPCR

Synchronized worms were collected by washing with M9 buffer and then subjected to sucrose density centrifugation to remove OP50 (*E*. *coli*) contamination. Total RNA was isolated from a frozen 1 ml aliquot (100 μl worm pellet dissolved in 1 ml TRIzol) by thawing and vigorous mixing according to the manufacturer’s instructions. The genomic DNA was digested by DNase I (Promega). Reverse transcription reactions were performed with iScript Reverse Transcription Supermix for RT-qPCR (Bio-Rad) with 1 μg of RNA. Fifty ng of cDNA was used for each real-time PCR reaction, which was performed with the iCycler IQ real-time PCR detection system (Bio-Rad). For the quantitative detection of *ncl-1*, fib-1, *act-1*, gfp and 26S rRNA transcripts, the following primer pairs were respectively used (the *act-1* transcript was simultaneously quantified as an internal control):

Qncl-1F’: 5’CAAATCGGAGGCGAGGGAGT3’

Qncl-1R’: 5’CGGAAGGAAGCGGTAGAGGTA3’

Qfib-1F’: 5’ CGTCGTTGGACCAGAAGGAAT 3’

Qfib-1R’: 5’ CACCGTTGCGAAGGAAGTTTT 3’

Qact-1F’: 5’GTGTGACGACGAGGTTGCCGCTCT3’

Qact-1R’: 5’GGTAAGGATCTTCATGAGGTAATC3’

QgfpF’: 5’ CATTGAAGATGGAAGCGTTC 3’

QgfpR’: 5’ ATAGTTCATCCATGCCATGT 3’

Q26S rRNAF’: 5’ GGAGTGCTTGTCTACTGCGAG 3’

Q26S rRNAR’: 5’ CCTCTGCACAGTCACAAGTG 3’

### Northern blot analysis

Synchronized late L4 worms grown at 15°C or 25°C as indicated were homogenized by a bead-beating homogenizer (FastPrep-24, MP Biomedicals) and total RNA was isolated by acid guanidinium thiocyanate-phenol-chloroform extraction [45]. Total RNA was subjected to 1.2% agarose-formaldehyde gel electrophoresis (5 μg/lane) and transferred to a Hybond-N+ membrane (GE Healthcare). DNA probes were generated from PCR products amplified from *C*. *elegans* genomic DNA and labeled with ^32^P-dCTP (Perkin Elmer, PK-BLU513H) by hexamer priming. Primers for generating the ribosomal RNA species and actin probes were performed as described by Voutev et al. [46]. Hybridization was carried out at 55°C in 0.36 M Na_2_HPO_4_, 0.14 M NaH_2_PO_4_, 1 mM EDTA, 10% SDS, 25% formamide and 0.1 mg/ml salmon sperm DNA. Washes were done at 55°C sequentially in 4× SSPE, 4% SDS and 0.1× SSC, 0.1% SDS. Membranes were exposed to Kodak BioMax MS film.

### Light microscopy and quantitative image analysis

To observe the FIB-1::GFP expression, embryos or young adult worms of *cguIs1* were pre-stained with WGA 555 (50 μg/ml) (Alexa Fluor 555 conjugate of wheat germ agglutinin, Invitrogen) at room temperature for 30 mins (embryos) or 4 h (worms) and collected by washing 3 times with M9 buffer. They were then mixed with embryos or worms of *ncl-1(e1942); cguIs1* in an equal ratio and mounted onto 5% agar pad (worms) or a chamber coverglass (embryo) (Thermo) for image acquisition. Bright field and fluorescence images were captured on an inverted or upright microscopy (Leica DMIRE2 and DM2500) using a 10×/NA 0.3 air immersion objective lens and a cool CCD (CoolSNAP K4, Roper Scientific). In order to distinguish the levels of GFP in the experimental and control embryos or worms under a same fluorescence microscope field, the average fluorescence intensity of different strains in the same images was measured using Metamorph 7.7.10.0 offline (Molecular Devices) and quantitatively determined by using Microsoft Excel software. For visualization of FIB-1::GFP expression and nucleolus size in worms, a upright microscope (Leica DM2500) with high-magnification, differential interference contrast (DIC) and fluorescence channels was used; images (shown in enlarged insets) were captured using a 63×/NA 1.4 oil immersion objective lens and a cool CCD (CoolSNAP K4). Metamorph 7.7.10.0 and Microsoft Excel software were used to measure the nucleolus size.

### Deconvolution microscopy

For visualization of GFP signals in the vulva and seam cells, transgenic worms at the L4 stage were paralyzed and mounted onto 5% agar pad for z-series image recording. The DIC and fluorescence signals were collected on a Deltavision deconvolution microscope (PersonalDV, Applied Precision) using a 60×/NA 1.4 oil immersion objective lens and a cool CCD (CoolSNAP HQ2, Roper Scientific). The Metamorph software version 7.7.10.0 offline was used in image analysis.

### Time-lapse images recording

Embryos of *cguIs1* or *ncl-1*(*e1942*); *cguIs1* as described above were plated onto a chamber coverglass for image acquisition. Phase contrast and fluorescence images were captured on an inverted microscope (Leica DMIRE2) using a 25×/NA 0.95 water immersion objective lens and an electron multiplying (EM) CCD (iXon ultra 897, Andor Technology). Images were recorded at 30s intervals and converted to pseudo-color using Metamorph software.

### Statistical analysis

Statistical analyses were performed with a two-tailed Student’s t-test for independent samples by using GraphPad Prism 5 software. P<0.05 was considered statistically significant.

## Supporting Information

S1 Fig Profiling of the *ncl-1* mRNA expression by RT-qPCR revealed a progressive decline in mRNA abundance from the embryo to and throughout the four larva stages, followed by subsequent up regulation in the adult. The bar graph shows values that were normalized to actin expression and averaged from three independent experiments, with error bars indicating standard error of mean (S.E.M.).
(TIF)

S2 Fig DIC microscopy of the *fib-1* larva (L1 stage, scale bar, 10 μm) revealed smaller nucleoli. Insets represent enlarged images of the boxed regions in the corresponding figures (scale bar, 2 μm).
(TIF)

S3 Fig Comparison between a pair of worms carrying a reporter with full-length *fib-1* 3' UTR showed significantly increased levels of FIB-1::GFP expression in the ncl-1 mutant background. *cguIs2* and *ncl-1(e1942); cguIs2* are respectively WGA-positive (indicated by numbers in red) and WGA-negative (in white). Insets in the lower panels represent enlarged versions of the boxed regions. Scale bar: 100 μm (upper panels) and 10 μm (lower panels).
(TIF)

S4 Fig Scheme of the *ncl-1* 3' UTR sequence showing complementary alignment with *let-7* and *mir-49*. Complementary nucleotides to the two microRNAs are indicated.
(TIF)

S5 Fig Related to Fig 5I. Contrasting effects of *ncl-1* and *let-7* on the expression of rRNA. Northern blot analysis of the expression of pre-rRNA and a processing intermediate in the indicated strains of worms, as shown in Fig 4g (*Actin* mRNA serves as a control).
(TIF)

S1 Movie Time-lapse recording of *cguIs1* embryos in phase contrast mode. (WMV)

S2 Movie Fluorescence time-lapse recording of *cguIs1* embryos as shown in S1 Movie. (WMV)

S3 Movie Pseudo-color time-lapse recording of *cguIs1* embryos as shown in S2 Movie. (WMV)

S4 Movie A z-series image recording of *cguIs8* that contains a *gfp*-reporter with the wild-type sequence of *ncl-1* 3' UTR [*ncl-1*::*gfp*::*ncl-1* 3' UTR (wt)]. (WMV)

S5 Movie A z-series image recording of *cguIs12* that contains a *gfp*-reporter with a mutated sequence of *ncl-1* 3' UTR [*ncl-1*::*gfp*::*ncl-1* 3' UTR (m)]. (WMV)

S1 Table Strains of worms generated in this study. (DOCX)
