# Supplementary figures and images for: A Genetic Cascade of let-7-ncl-1-fib-1 Modulates Nucleolar Size and rRNA Pool in Caenorhabditis elegans
Source: PLoS Genet. 2015 Oct 22;11(10):e1005580. doi: 10.1371/journal.pgen.1005580 (PMC4619655; doi:10.1371/journal.pgen.1005580)

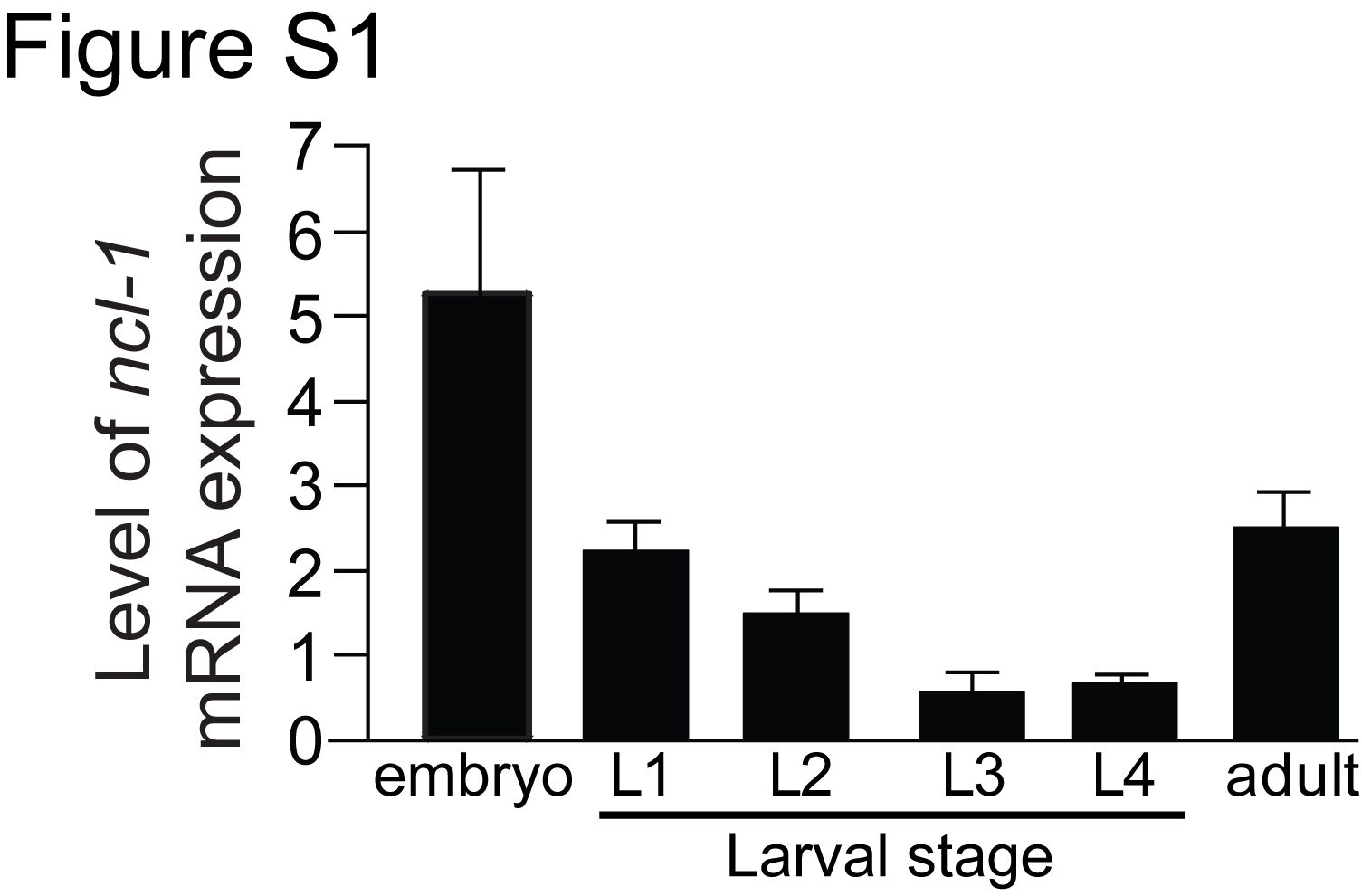

Supplement: S1 Fig — The bar graph shows values that were normalized to actin expression and averaged from three independent experiments, with error bars indicating standard error of mean (S.E.M.). (TIF) [file pgen.1005580.s001.tif]

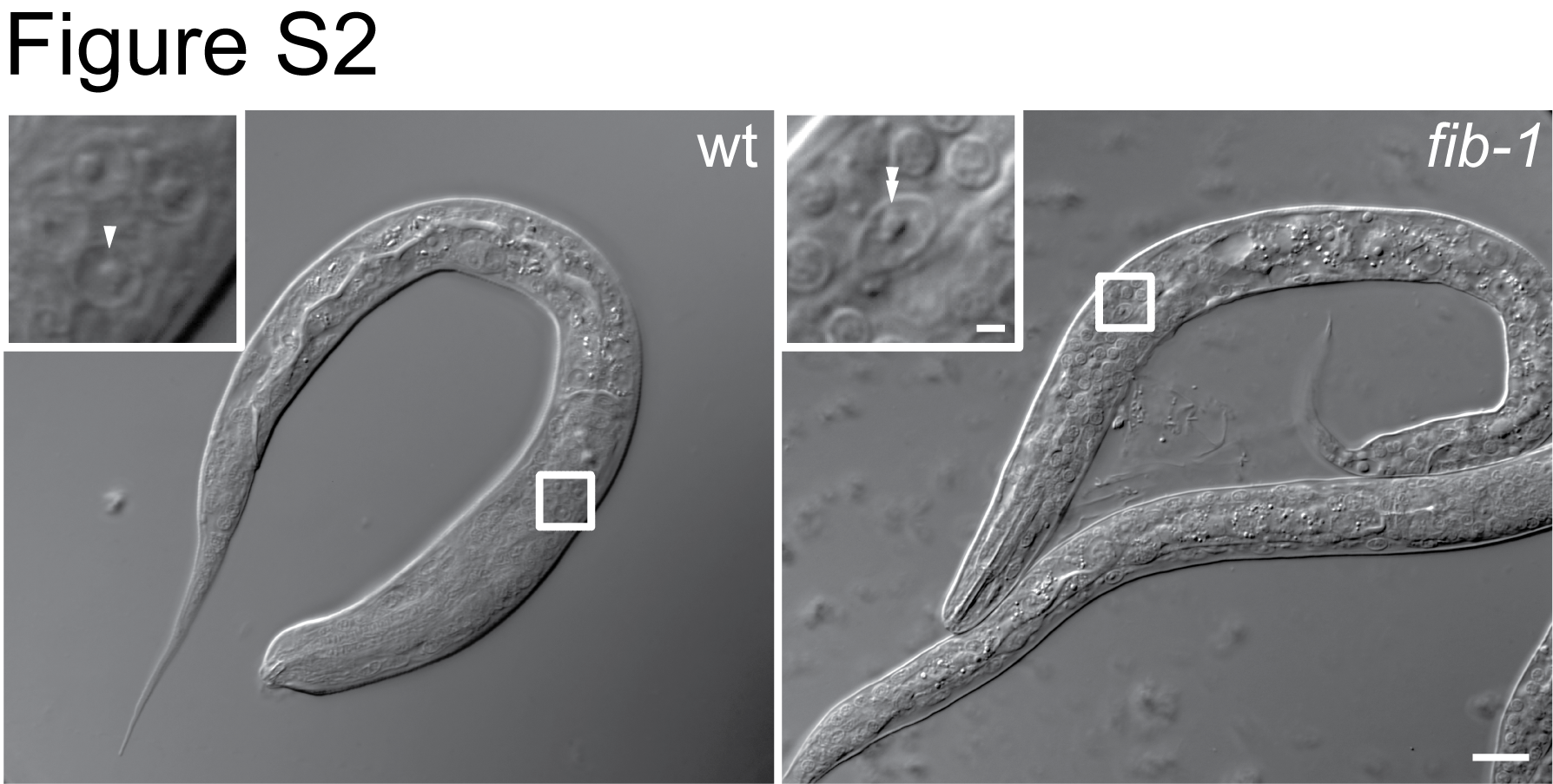

Supplement: S2 Fig — Insets represent enlarged images of the boxed regions in the corresponding figures (scale bar, 2 μm). (TIF) [file pgen.1005580.s002.tif]

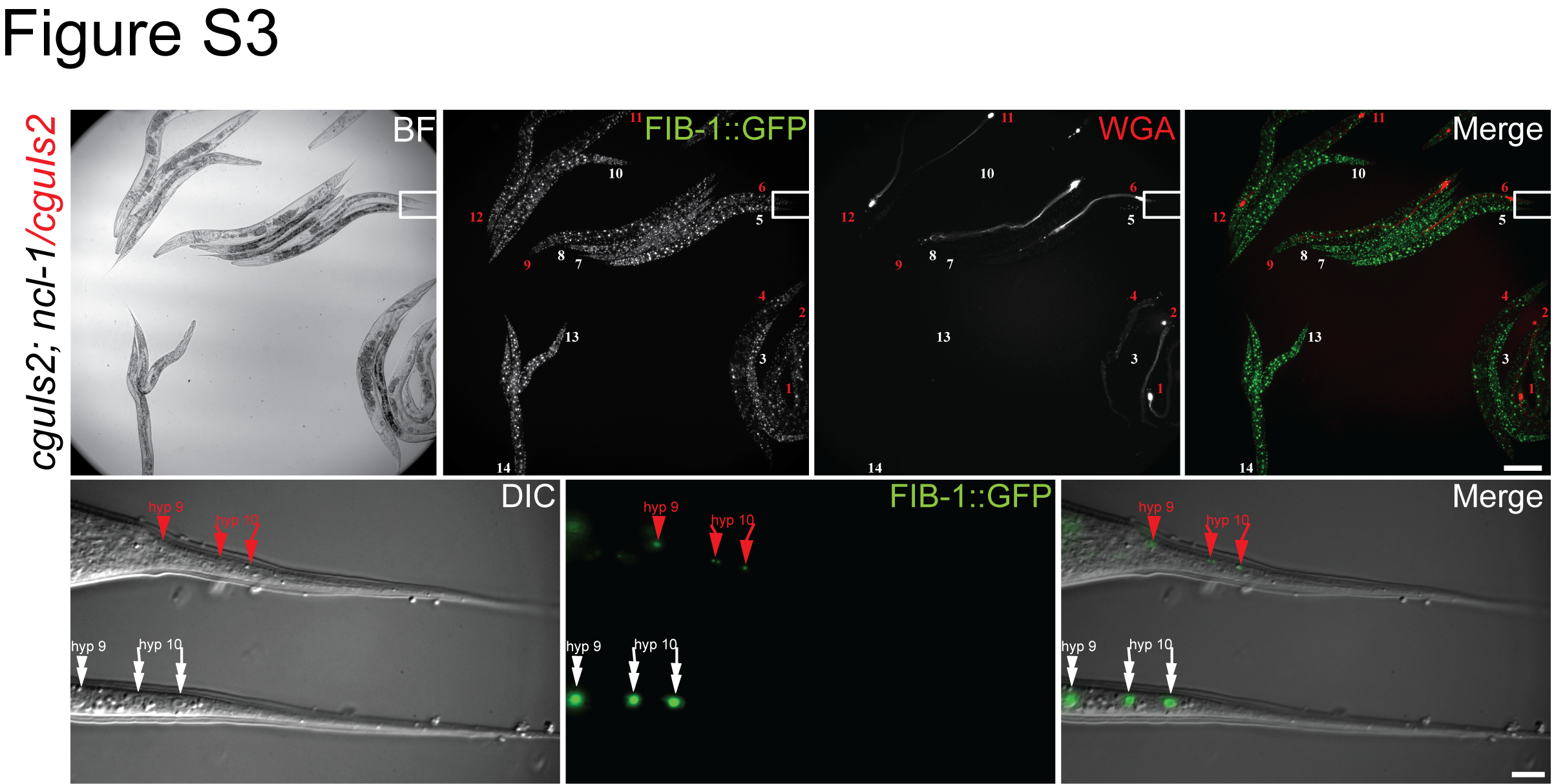

Supplement: S3 Fig — cguIs2 and ncl-1(e1942); cguIs2 are respectively WGA-positive (indicated by numbers in red) and WGA-negative (in white). Insets in the lower panels represent enlarged versions of the boxed regions. Scale bar: 100 μm (upper panels) and 10 μm (lower panels). (TIF) [file pgen.1005580.s003.tif]

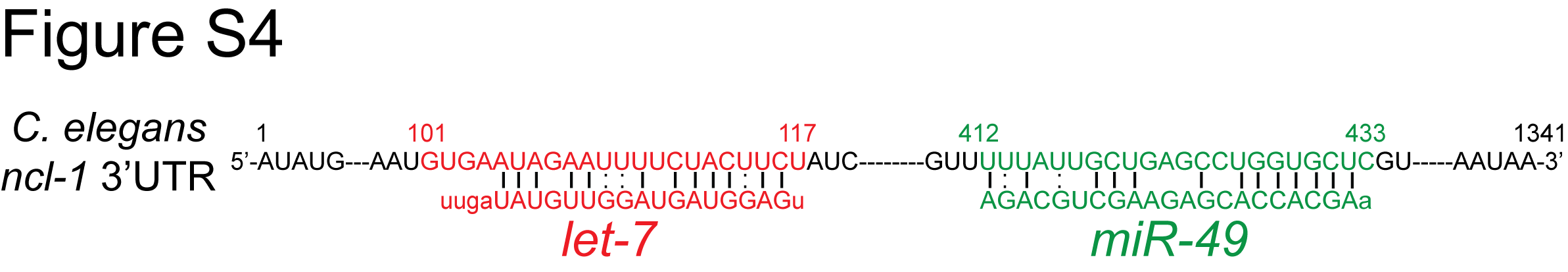

Supplement: S4 Fig — Complementary nucleotides to the two microRNAs are indicated. (TIF) [file pgen.1005580.s004.tif]

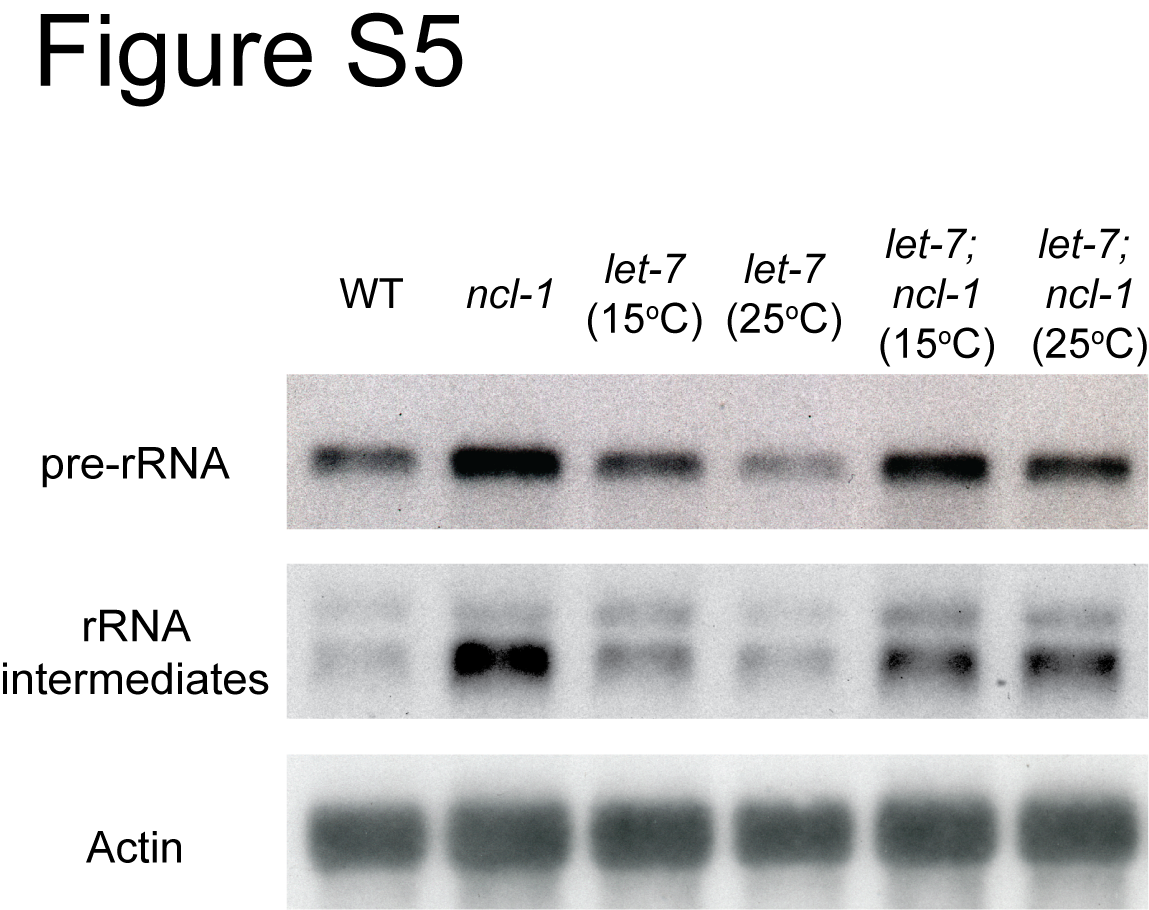

Supplement: S5 Fig — Northern blot analysis of the expression of pre-rRNA and a processing intermediate in the indicated strains of worms, as shown in Fig 4g (Actin mRNA serves as a control). (TIF) [file pgen.1005580.s005.tif]
