# Supplementary material for: A Genetic Cascade of let-7-ncl-1-fib-1 Modulates Nucleolar Size and rRNA Pool in Caenorhabditis elegans
Source: PLoS Genet. 2015 Oct 22;11(10):e1005580. doi: 10.1371/journal.pgen.1005580 (PMC4619655; doi:10.1371/journal.pgen.1005580)
Supplement: S1 Table — (DOCX) [file pgen.1005580.s011.docx]

**S1 Table Strains of worms** **generated in this study**

| **Strain** | **Genotype** | **Integration Method** |
| --- | --- | --- |
| SJL1 | *cguIs1* [*P_fib-1_::fib-1::gfp::3' UTR _fib-1_*] | MI/UV* |
| SJL2 | *cguIs2* [*P_fib-1_::fib-1::gfp::3' UTR _fib-1_*] | MI/UV* |
| SJL6 | *cguIs6* [*P_ncl-1_::gfp::3' UTR_ncl-1_*] | bombardment |
| SJL7 | *cguIs7* [*P_ncl-1_::gfp::3' UTR_ncl-1_*] | bombardment |
| SJL8 | *cguIs8* [*P_ncl-1_::gfp::3' UTR_ncl-1_*] | bombardment |
| SJL9 | *cguIs9* [*P_ncl-1_::gfp::3' UTR_ncl-1_*] | bombardment |
| SJL10 | *cguIs10* [*P_ncl-1_::gfp::3' UTR_ncl-1_*_(m)_] | bombardment |
| SJL11 | *cguIs11* [*P_ncl-1_::gfp::3' UTR_ncl-1_*_(m)_] | bombardment |
| SJL12 | *cguIs12* [*P_ncl-1_::gfp::3' UTR_ncl-1_*_(m)_] | bombardment |
| SJL31 | *cguIs16* [*P_fib-1_::fib-1::gfp::3' UTR_unc-54_*] | MI/UV* |
| SJL32 | *cguIs17* [*P_fib-1_:: fib-1::gfp::3' UTR_unc-54_*] | MI/UV* |
| SJL33 | *cguIs18* [*P_fib-1_:: fib-1::gfp::3' UTR_unc-54_*] | MI/UV* |
| SJL34 | *cguIs19* [*P_fib-1_:: fib-1::gfp::3' UTR_unc-54_*] | MI/UV* |
| SJL14 | *ncl-1(e1942); cguIs1* | crossing |
| SJL15 | *ncl-1(e1942); cguIs2* | crossing |
| SJL38 | *ncl-1(e1942); cguIs19* | crossing |
| SJL39 | *let-7(n2853); ncl-1(e1942)* | crossing |
| SJL117 | *cguEx18* [P*_fib-1_:: fib-1::gfp::*3' UTR*_fib-1_*_(m)_] | MI/UV* |
| SJL118 | *ncl-1(e1942); cguEx18* | crossing |

*microinjection followed by UV irradiation
